# Long noncoding RNAs in the initiation, progression, and metastasis of hepatocellular carcinoma

**DOI:** 10.1016/j.ncrna.2017.11.001

**Published:** 2017-12-05

**Authors:** Johanna K. DiStefano

**Affiliations:** Program in Epigenetics and Fibrosis, Translational Genomics Research Institute, 445 N 5th Street, Phoenix, AZ 85004, United States

**Keywords:** Long noncoding RNAs, lncRNAs, Noncoding RNA, Hepatocellular carcinoma, HULC, HOTAIR

## Abstract

Hepatocellular carcinoma (HCC) is the third leading cause of cancer-related death worldwide. Despite awareness of risk factors for the development of HCC and advances in the diagnosis and clinical management of the disease, the molecular mechanisms underlying hepatocarcinogenesis remain poorly understood. Recent experimental studies provide strong evidence that long noncoding RNAs (lncRNAs), non-protein-coding transcripts with lengths >200 basepairs, contribute to the pathogenesis of numerous human diseases. Over the past decade, a role for lncRNAs in the initiation, progression, and metastasis of HCC has likewise emerged and developed into a highly active area of research. Although many lncRNAs appear to be dysregulated in HCC, extensive functional characterization has been performed on only a small proportion of these candidates to date. This review summarizes select lncRNAs that have been shown to wield functional relevance in the initiation, progression, or metastasis of HCC, focusing on the specific mechanisms by which lncRNA effects might be linked to clinical manifestations of the disease. In addition, an overview of circulating lncRNAs that have been identified as potential biomarkers for the diagnosis and prognosis of HCC is provided.

## 1 Introduction

Hepatocellular carcinoma is the most common form of liver cancer, and in the United States, the disease has an annual incidence of at least 6 per 100,000 [1] and represents the fastest rising cause of cancer-related death [2]. Despite recent improvements in diagnostic methods and surgical techniques, the five-year survival rate for advanced HCC remains dismal [3]. Risk factors for the initiation of HCC include viral infection, nonalcoholic fatty liver disease, alcohol overconsumption, aflatoxin, and genetic factors; the majority of these conditions also contribute to the development of hepatic cirrhosis, which promotes HCC formation [2]. In fact, HCC is the most common cause of mortality in cirrhotic patients [4]. Although the risk factors for HCC are well characterized, the molecular mechanisms underlying the malignant transformation of hepatocytes are still incompletely understood [5].

There are approximately 60,000 noncoding RNAs in the human genome, 68% of which are long noncoding RNAs or lncRNAs [6]. Excellent reviews of the origin, biogenesis, and characteristics of lncRNAs, including the manner in which they are distinguished from other ncRNAs, can be found elsewhere [7], [8], [9], [10], and will therefore not be reiterated here. Because lncRNAs are typically expressed at much lower levels than protein-coding transcripts (i.e., messenger RNAs), these transcripts were considered to be transcriptional noise when first discovered in the early 1990s [11], [12], [13]. However, unprecedented advances in both high-throughput sequencing technologies and computational methods have fostered the identification and annotation of a number of biologically relevant lncRNAs. At the same time, a substantial amount of experimental evidence supporting a role for lncRNAs in biological processes underlying disease pathophysiology is accumulating at a rapid pace in the scientific literature.

Over the past decade, a role for lncRNAs in the initiation, progression, and metastasis of HCC has emerged and developed into a highly active area of research. Although many lncRNAs appear to be dysregulated in HCC [14], [15], [16], [17], [18], [19], [20], [21], [22], [23], only a small proportion of these have so far been extensively characterized. This review summarizes select lncRNAs that have been shown to wield functional relevance in the initiation, progression, or metastasis of HCC, focusing on the specific mechanisms by which lncRNA effects might be linked to clinical manifestations of the disease. In addition, an overview of circulating lncRNAs that have been identified as potential biomarkers for the diagnosis and prognosis of HCC is provided.

## 2 lncRNAs associated with initiation, progression, and metastasis of HCC

A number of studies have investigated lncRNA signatures associated with HCC. Microarray profiling analysis of resected tumor tissues and matched adjacent non-tumor tissues from 29 HCC patients (22 HBV-positive and 3 HCV-positive) revealed 659 differentially expressed lncRNAs [24]. Of these, five lncRNAs were randomly selected for validation using quantitative reverse-transcription PCR (qRT-PCR), four of which were found to be significantly downregulated in tumor tissue: TCONS_00018278, AK093543, D16366, and ENST00000501583. An independent microarray analysis performed using three pairs (two HBV-positive and one HCV-positive) of HCC and adjacent non-tumor tissues reported differential expression of 214 lncRNAs [25]. Fifty of these lncRNAs were subsequently selected for validation with qRT-PCR in three sets of HCC tissues, of which expression patterns of 40 lncRNAs matched the microarray results. Dysregulated expression of eight lncRNAs (BC017743, ENST00000395084, NR_026591, NR_015378, NR_024284, NR_027151, AK056988, and uc003yqb.1) was confirmed in an expanded sample of nineteen matched HCC-non-tumor pairs. Both studies were limited by small sample sizes and differential distribution of patients infected with HBV or HCV, which may explain, in part, the lack of overlap between findings. A third study analyzed expression data from 372 HCC patients from The Cancer Genome Atlas (TCGA) and the NCBI GEO omnibus, and identified 177 cancer-specific lncRNAs, 41 of which were associated with gender, race, tumor grade, tumor stage, and TNM staging [26]. Using the univariate Cox proportional hazards regression model, CECR7, LINC00346, MAPKAPK5-AS1, LOC3338651, FLJ90757, and LOC283663 were found to be associated with overall survival. In addition to the identification of novel lncRNA candidates using a well-powered sample, this study also provided the first evidence of crosstalk among lncRNAs, miRNAs, and mRNAs in the progression of HCC, suggesting that further investigation of relationships between different coding and noncoding transcripts is warranted.

In addition to global lncRNA profiling, a number of studies have examined expression levels of individual lncRNAs. Among these findings, dysregulated expression of ANRIL [27], GAS5 [28] and PCAT-1 [29] has been associated with poor prognosis of HCC. An overview of lncRNAs dysregulated in HCC tumor tissue can be found elsewhere [30], and a comprehensive list of lncRNAs associated with HCC initiation, progression, and metastasis is shown in Table 1. Despite the accumulating evidence supporting dysregulated lncRNA expression in HCC, very few lncRNAs have thus far been functionally characterized with respect to the molecular underpinnings of disease pathogenesis. However, two lncRNAs, HULC and HOTAIR, have been relatively well studied and an overview of the roles played by these candidates in biological processes relevant to HCC is presented below.

**Table 1:** lncRNAs associated with HCC initiation, progression, and metastasis in humans.

lncRNA	Biological association	Ref
AFAP-AS1	Promotes HCC cell proliferation and metastasis	[73], [74]
AOC4P	Modulates cell proliferation, migration and invasion via EMT inhibition	[75]
ANRIL	Regulates HCC cell proliferation and apoptosis	[76]
CARLo-5	Promotes HCC cell proliferation, invasion, and metastasis	[77]
CCAT1	Promotes HCC cell proliferation and invasion	[78], [79], [80]
CCHE1	Promotes HCC progression	[15]
DANCR	Promotes HCC cell proliferation and metastasis; increases stemness features of HCC cells	[81], [82]
DBH-AS1	Promotes HCC cell proliferation	[83]
FTX	Inhibits HCC cell growth and metastasis	[84]
GAS5	Promotes HCC cell proliferation and invasion through regulation of vimentin	[85], [86]
GPC3-AS1	Promotes HCC cell proliferation and migration. Activates GPC3 to promote HCC progression	[87]
H19	Enhances tumorigenicity	[88]
HEIH	Facilitates tumor growth through enhancer of zeste homolog 2	[89]
HNF1A-AS1	Promotes autophagy and oncogenesis in HCC	[90]
HOTAIR	Promotes HCC cell migration and invasion and activates autophagy	[19], [44], [45], [46], [47], [49]
HOTTIP	Promotes HCC cell proliferation and tumorigenicity	[91], [92]
HOXA-AS2	Promotes HCC cell proliferation and invasion, regulates apoptosis, and causes tumorigenicity in nude mice	[93]
HULC	Promotes HCC cell proliferation, triggers autophagy, and enhances tumor angiogenesis	[37], [39], [94], [95]
Linc00152	Promotes HCC cell proliferation through activation of mTOR signaling pathway	[96]
Linc01225	Promotes HCC cell proliferation and invasion	[97]
Linc00974	Promotes HCC cell proliferation and invasion	[98]
Linc-cdh4-2	Inhibits migration and invasion of HCC cells via R-cadherin pathway	[99]
lincRNA-p21	Inhibits HCC invasion and metastasis	[14], [100]
LOC90784	Promotes HCC cell proliferation and invasion	[101]
lncRNA-ATB	Promotes invasion-metastasis cascade in HCC	[102]
lncCAMTA1	Promotes HCC cell proliferation, cancer stem cell-like properties, and tumorigenesis.	[103]
lncTCF7	Promotes HCC aggressiveness through EMT activation, promotes cancer stem cell self-renewal and tumor propagation	[104], [105]
MALAT1	Promotes tumor progression	[106]
NEAT1	Promotes HCC cell proliferation	[107]
PANDAR	Promotes tumorigenesis in HCC	[108]
PCAT1	Promotes HCC cell proliferation and migration; inhibits apoptosis	[109]
PlncRNA-1	Promotes HCC ell proliferation, migration, and invasion via EMT signaling	[110]
PVT1	Promotes HCC cell proliferation and cancer stem cell-like properties	[111]
SNHG1	Promotes tumorigenesis in HCC	[112], [113]
SNHG-003	Promotes HCC cell proliferation	[114]
Sox2ot	Promotes HCC metastasis	[115]
SPRY4-IT1	Promotes tumor cell proliferation and invasion	[116]
TP73-AS1	Modulates HCC cell proliferation	[117]
TUG1	Promotes HCC cell growth and apoptosis through KLF2 sillencing	[118]
TUSC7	Suppressed EMT in HCC	[119]
Uc001kfo	Promotes proliferation, metastasis and EMT in HCC cells by targeting alpha-SMA	[120]
Uc-134	Promotes HCC cell proliferation, invasion, and metastasis	[121]
Uc-338	Promotes HCC cell proliferation and induces cell cycle progression	[122]
UCA1	Promotes HCC cell growth, tumorigenesis, and HCC progression	[123], [124]
UFC1	Promotes HCC cell proliferation and cell-cycle progression and inhibits apoptosis	[125]
Unigene56159	Promotes cell migration/invasion and EMT in HCC	[126]
URHC	Regulates HCC cell proliferation and apoptosis via ERK/MAPK signaling pathway	[127]
XIST	Inhibits HCC cell proliferation and metastasis by targeting miR-92b	[128]
ZEB-AS1	Promotes tumor growth and metastasis	[129], [130]
ZFAS1	Promotes metastasis in HCC	[131]
ZNFX1-AS1	Modulates HCC cell proliferation	[17]

### 2.1 HULC (highly upregulated in liver cancer)

HULC was first identified as a result of a genome-wide search for novel transcripts associated with the molecular pathogenesis of HCC [31]. HCC-specific cDNA libraries and tissue samples from 46 HCCs were analyzed, and one transcript was found to be significantly upregulated (33-fold) in 76% of tumors compared to a non-neoplastic pool of liver samples. Expression levels of this transcript were low in normal tissue and not significantly increased in other neoplastic tissues, suggesting that upregulation of this transcript was specific to HCC; thus this previously unannotated transcript was given the name “highly upregulated in liver cancer”. In a study of 38 patients with HCC, HULC levels were also associated with clinical stage, intrahepatic metastases, HCC recurrence, and postoperative survival [32]. HULC knockdown in hepatoma cell lines resulted in dysregulated expression of many genes, including several with an established role in liver cancer; however, no significant sequence homology was observed between HULC and these potential target genes, indicating that HULC-mediated effects are probably exerted via direct RNA-RNA interactions.

The HULC transcript is a 482 bp, spliced, polyadenylated ncRNA that localizes to the cytoplasm and co-purifies with ribosomes of carcinoma cells [31]. HULC shows evolutionary conservation in primates, although neither the mouse nor rat genome appears to have a HULC homologue [31]. A cAMP response element-binding (CREB) site in the HULC proximal promoter region was shown to be important for transcriptional activity in liver cancer [33]. In addition, HULC RNA can sequester miRNAs to regulate gene expression. For example, HULC was found to bind miR-372, leading to reduced expression of its target gene, protein kinase cAMP-activated catalytic subunit beta (PRKACB) [33].

Chronic infection with the hepatitis B virus (HBV) is associated with HCC development [34]. Levels of HBV X protein (HBx), a protein produced by HBV, are elevated in liver cells from HCC patients [35] and the HBx protein activates genes associated with cellular growth [36]. In an analysis of 33 HCC specimens, expression levels of HBx and HULC were positively correlated [37]. Knockdown of HBx expression corresponded with decreased HULC levels in hepatoma cells, while HBx overexpression resulted in a dose-dependent increase in HULC. Together, these results suggest that HBx regulates HULC expression in liver cells. Further, HULC downregulated expression of p18, a tumor suppressor gene located in close proximity to HULC, leading to proliferation of hepatoma cells. In hepatoma cells, HBx was found to downregulate p18, which was reversed with HULC knockdown, implicating a mechanism in which HBx-mediated upregulation of HULC promotes the proliferation of hepatoma cells through downregulation of p18 [37].

Depletion of insulin growth factor 2 mRNA-binding protein 1 (IGF2BP1) corresponded with increased half-life and steady state transcript levels of HULC [38]. CNOT1 (C-C motif chemokine receptor 4–NOT transcription complex subunit 1), a major component of the cytoplasmic RNA decay machinery, was identified as a novel interaction partner for IGF2BP1, and depletion of CNOT1 also resulted in increased half-life and expression of HULC. Thus, the main finding of this study was the characterization of IGF2BP1 as an adaptor protein capable of recruiting the CNOT1 complex and initiating the degradation of HULC [38].

Additional studies have served to shed light on the role of HULC in HCC development. For example, HULC was found to promote tumor angiogenesis by upregulating sphingosine kinase 1 (SPHK1) [39], an enzyme that generates sphingosine phosphate, which is known to contribute to cell survival, proliferation, differentiation, and angiogenesis [40], [41], [42]. Through a series of experiments, HULC was shown to increase expression of the E2F1 transcription factor, which binds to the SPHK1 promoter. HULC was further found to sequester miR-107, a known regulator of E2F1, resulting in a cascade of upregulated E2F1, increased SPHK1 activation, and eventually, tumor angiogenesis. These findings provided novel insight into molecular mechanisms underlying tumor angiogenesis in HCC.

HULC was also recently shown to enhance epithelial-mesenchymal transition (EMT), which contributes to tumor metastasis and recurrence in HCC via a signaling pathway involving zinc finger E-box binding homeobox 1 (ZEB1) and miR-200a-3p [32]. HULC was found to sequester miR-200a-3p, leading to increased levels of ZEB1, which corresponded to stabilized EMT. Together, these results reveal a potentially novel mechanism by which HULC mediates pathophysiology in HCC.

### 2.2 HOTAIR (Homeobox [HOX] transcript antisense RNA)

Although HOTAIR was initially identified under the auspices of an ultra-high resolution tiling microarray designed to interrogate the transcriptional and epigenetic landscape of HOX loci in primary human fibroblasts [43], its association with HCC was first reported in a study of 110 HCC samples using qRT-PCR [44]. In that study, HOTAIR was expressed at significantly higher levels in HCC tumor compared to adjacent non-cancerous tissue and was an independent prognostic factor for predicting HCC recurrence in liver transplantation patients. In patients who exceeded the criteria for liver transplantation (single HCC ≤ 5 cm or up to 3 HCCs ≤ 3 cm), high HOTAIR levels were associated with a shorter recurrence-free survival. Functional knockdown of HOTAIR expression in liver cancer cells corresponded with decreased cell viability and invasion, increased TNF-α induced apoptosis, and heightened sensitivity to chemotherapeutic agents [44]. A second study published in the same year reported upregulation of HOTAIR in surgically resected tumor tissue from 63 HCC patients compared with adjacent non-tumor tissues [45]. Patients with high levels of HOTAIR expression showed an elevated risk of post-hepatectomy recurrence and lymph node metastasis. Similar to the previous study [44], HOTAIR knockdown corresponded with decreased HCC cell proliferation, and was also found to be associated with reduced levels of matrix metalloproteinase-9 (MMP9) and vascular endothelial growth factor protein (VEGF), both of which contribute to cell motility and metastasis. These results were replicated in additional studies, providing strong support for HOTAIR in biological mechanisms underlying HCC progression [45], [46], [47].

Expression profiling of coding transcripts following HOTAIR knockdown in HCC cells resulted in the dysregulation of 296 genes, including upregulation of QKI, KH domain containing RNA binding (QKI), CD82, and RNA binding motif protein 38 (RBM38); increased transcript and protein levels of these three genes were further validated by qRT-PCR analysis and western blotting, respectively [46]. However, only levels of RBM38, but not QKI and CD82, were dysregulated in HCC samples compared to adjacent non-tumor paired samples, leading the research team to focus on this gene as being functionally relevant to HOTAIR. Concordant with this finding, knockdown of HOTAIR expression in HepG2 and Bel-7402 cells resulted in increased transcript and protein levels of RBM38, and corresponded with reduced HCC cell migration and invasion, which was specifically rescued by RBM38 downregulation. These findings showed that HOTAIR promotes HCC cell migration and invasion through inhibition of RBM38. A similar investigation of HOTAIR depletion showed downregulated expression of Wnt and β-catenin [48], providing evidence that HOTAIR may affect HCC progression through multiple signaling pathways.

In an investigation of HOTAIR in HCC cell models and a xenograft mouse model, HOTAIR was shown to negatively regulate miR-218 expression in HCC through a promoter regulatory axis involving EZH2-targeting-miR-218-2 [49]. HOTAIR knockdown in vitro was found to inhibit HCC cell viability and induce G1-phase arrest, while in vivo depletion of HOTAIR was shown to suppress tumorigenicity by disinhibiting miR-218 expression. The Bmi-1 oncogene was identified as a functional target of miR-218, and the main downstream targets signaling, P16 (Ink4a) and P14 (ARF), were activated in HOTAIR-suppressed tumorigenesis. In primary human HCC specimens, HOTAIR and Bmi-1 were concordantly upregulated whereas miR-218 was downregulated in these tissues. Furthermore, HOTAIR was inversely associated with miR-218 expression and positively correlated with Bmi-1 expression in these clinical tissues.

Additional studies aimed at understanding the molecular mechanisms by which HOTAIR impacts tumor cell development have been recently undertaken. For example, in one study, overexpression of HOTAIR was found to promote activation of autophagy in HCC cell lines, while depletion of the lncRNA suppressed this pathway [19]. In this model, upregulated HOTAIR corresponded with increased expression autophagy-related 3 (ATG3) and ATG7 expression in HCC cells, suggesting a novel pathway promoting HCC cell proliferation. In another study, RNA-protein complexes formed with HOTAIR were regulated by the RNA helicase DEAD box protein 5 (DDX5), and associated with HBV biosynthesis and poor prognosis in HBV-mediated liver cancer [50]. Specifically, DDX5 was shown to interact with suppressor of zeste 12 homolog (SUZ12), the core subunit of the chromatin-modifying polycomb repressive complex 2 (PRC2), and in association with HOTAIR, repressed the transcription of epithelial cell adhesion molecule (EpCAM) and three pluripotency genes.

In an investigation of HOTAIR, forkhead box C1 (FOXC1), and miR-1, levels of HOTAIR and FOXC1 were increased, while levels of miR-1 were decreased in HCC tissues and HepG2 cells compared to normal liver cells and adjacent non-tumor tissues [51]. Overexpression of HOTAIR in the immunodeficient nude mouse model (nu/nu) resulted in enhanced HCC cell proliferation and progression of tumor xenografts. Functional characterization studies showed that FOXC1 binds to an upstream region of HOTAIR and activates its expression in HCC cells, while HOTAIR negatively regulated miR-1 expression. Results from this work suggested that HOTAIR is a FOXC1-activated driver of malignancy, which acts in part through the repression of miR-1.

Most recently, Zhou et al. [52] demonstrated that HOTAIR knockdown corresponded with reduced cell proliferation and increased G0/G1 cell cycle arrest in Huh7 cells. HOTAIR knockdown was also associated with reduced levels of cyclin D1 (CCND1) transcript and protein, as well as phosphorylated signal transducer and activator of transcription 3 (STAT3), which led to an even greater decrease in CCND1 expression. These results provided evidence that HOTAIR may impact HCC cell proliferation through regulation of cell cycle, STAT3 activity, and CCND1 expression.

Since its annotation in 2007, HOTAIR has emerged as a novel prognostic marker for HCC. While a number of studies have indicated multiple pathways by which HOTAIR may affect HCC cell proliferation and invasion, further investigation of the molecular mechanisms underlying dysregulated HOTAIR expression and the manner in which the lncRNA promotes HCC progression are necessary to nominate its use as a potential therapeutic target in the treatment of HCC.

## 3 lncRNAs as biomarkers for HCC diagnosis and prognosis

A number of different treatment options are available for HCC patients, including surgical resection, liver transplantation, ablation (radiofrequency, cryoablation, and percutaneous ethanol injection), transcatheter arterial chemoembolization, radioembolization, and chemotherapy with the multi-kinase inhibitor sorafenib [53]. Selection of therapeutic intervention depends on many factors such as tumor stage, the ability of the patient to tolerate surgery, hepatic function, and availability of organs, in the case of liver transplantation; however, in all cases, early diagnosis is key for optimal survival rates. Due to an absence of early-stage symptoms, HCC often remains undiagnosed until advanced stages of disease, which limits opportunities for successful intervention, leading to poor prognosis and reduced five-year survival rate [54]. It is widely recognized that improved screening methods are critical for early diagnosis of HCC and enhanced survival rates for HCC patients.

A variety of imaging techniques are available for HCC screening, each presenting with unique advantages and disadvantages [55]. For example, computed tomography (CT) is an easily accessible imaging modality useful for clinical routine work-up of HCC [55]. However, while CT and magnetic resonance imaging (MRI) both yield acceptable ranges of sensitivity (55%–91%) and specificity (77%–96%), the relatively high cost and the potential harm arising from radiation exposure limit their use for large-scale screening and routine surveillance [56]. On the other hand, liver ultrasound, which represents the primary surveillance modality for HCC, has a sensitivity of ∼60% and a specificity of 85–90% [57], but is less accurate in obese patients or in the presence of a nodular liver [58]. In addition, ultrasound is associated with decreased detection compared to CT and MRI, albeit with a lower false positive rate [59].

In addition to imaging screening modalities, a number of serum biomarkers are available for the detection of HCC [60]. These include alpha-fetoprotein (AFP), gamma-glutamyl transferase (GGT), des-γ-carboxyprothrombin (DCP), glypican-3 (GPC3), osteopontin, and others, all of which having varying levels of sensitivity and specificity [54], [61]. Of these, AFP is the most widely used serum biomarker for the diagnosis and monitoring of HCC; however, the specificity of this glycoprotein is limited. For example, normal levels of AFP are present in ∼30% of HCC patients at the time of diagnosis and remain low even with advanced stages of disease [62]. Likewise, the range of AFP values in HCC is quite wide, from normal concentrations to >100,000 ng/mL [62], and non-cancer liver diseases such as chronic hepatitis or cirrhosis are associated with increased AFP levels [63]. AFP >400–500 ng/ml is considered diagnostic for HCC, although fewer than half of patients present with values in that range [62].

To be clinically useful, biomarkers should demonstrate high sensitivity and specificity for HCC, have concentrations that reflect changes in tumor progression and prognosis, and be easily and non-invasively detectable in body fluids. LncRNAs have emerged as diagnostic biomarkers for human diseases, particularly cancer [64], [65] and cardiovascular disease [66], due to their stability in biofluids. In HCC, hepatic expression levels of many lncRNAs have been found to be dysregulated in tumor samples relative to normal tissue; however, the number of lncRNAs showing differences in circulating concentrations is relatively small [30]. A summary of differentially expressed, circulating lncRNAs is shown in Table 2, and a detailed discussion of several potential biomarkers, including HULC (highly upregulated in liver cancer), MALAT1 (metastasis-associated lung adenocarcinoma transcript 1), and UCA1 (urothelial-associated carcinoma 1), is presented below.

**Table 2:** lncRNAs implicated as circulating biomarkers for the diagnosis and prognosis of HCC.

lncRNA	lncRNA name	Major findings	Ref
DANCR	Differentiation antagonizing non-protein coding RNA	High plasma levels differentiated HCC patients from healthy volunteers and individuals with non-cancerous liver disease; high plasma levels associated with microvascular invasion and liver capsule invasion in HCC patients	[81]
DGCR5	DiGeorge syndrome critical region gene 5	Low serum levels associated with poor cancer-specific survival and were independent negative prognostic factor for HCC	[132]
HEIH	High Expression in HCC	Plasma levels increased in HCC patients compared to healthy individuals	[30]
HULC	Highly upregulated in liver cancer	Plasma levels increased in HCC patients compared to healthy individuals	[67], [68]
LINC00152		Plasma levels increased in HCC patients compared to healthy individuals	[68]
LINC01225		Plasma levels increased in HCC patients compared to healthy individuals	[97]
MALAT1	Metastasis-associated lung adenocarcinoma transcript 1	Plasma levels increased in HCC patients compared to healthy individuals	[70]
PVT1 & uc022mbe.2	Plasmacytoma variant translocation 1	Combined lncRNA signature could distinguish HCC patients from healthy individuals and was associated with associated with tumor size, Barcelona Clinic Liver Cancer stage, and serum bilirubin	[20]
RP11-160H22 &XLOC_014172 & LOC149086		Plasma levels of three-lncRNA signatures discriminated HCC patients from healthy individuals; XLOC_014172, LOC149086, showed a higher expression in HCC patients with metastasis	[133]
SPRY4-IT1	SPRY4 intronic transcript 1	Plasma levels could distinguish HCC patients from healthy controls	[134]
uc001ncr & AX800134		Able to accurately diagnose HBV-positive HCC and early HCC	[135]
uc003wbd & AF085935		Serum levels higher in HCC and HBV patients compared with normal controls	[136]
UCA1	Urothelial carcinoma-associated 1	Higher expression in HCC, associated with higher tumor grade, large tumor size, positive vascular invasion, and advanced TNM stage	[71], [72]
WRAP53	WD repeat containing, antisense to TP5	Higher serum levels in HCC and significant independent prognostic marker in relapse-free survival	[71]

### 3.1 HULC

In the initial work identifying HULC as a ncRNA upregulated in HCC, a pilot study comprised of four HCC patients, ten patients with hepatic cirrhosis, and nine healthy individuals found that peripheral blood levels of HULC RNA were substantially higher in patients with HCC compared to individuals with no evidence of liver disease, and that for two of the HCC patients, blood levels of HULC mirrored those in surgically resected tumors [31]. These findings were subsequently corroborated in a larger study of 30 HCC patients and 20 healthy individuals, in which HULC was detected in plasma samples of 63% of the HCC patients [67]. In these patients, HULC detection frequencies increased in accord with Edmondson and Steiner grade, an established method of histological classification. HULC was also detected more frequently in HCC patients with HBV versus those without HBV (90% vs. 25%), although the implications of this finding are not yet clear. In an investigation of eight lncRNA candidates in 24 HCC patients and an equal number of healthy individuals, HULC levels were significantly higher in HCC patients and were correlated with tumor size and tumor capsular [68]. Receiver operating characteristic (ROC) curve analysis for HULC was 0.78, which increased when combined with AFP concentration [68]. Together, these findings demonstrated that circulating HULC levels are elevated in HCC patients, are reflective of neoplasm expression levels, and are associated with tumor aggressiveness and progression. While the prognostic power of HULC requires further substantiation by longitudinal analysis in prospective studies, these reports provide a significant step toward establishing the utility of HULC expression as a prognostic indicator for HCC.

### 3.2 MALAT1

In an analysis of gene expression from HCC, hepatoblastomas, tissue adjacent to HCC tumors, and normal tissue, MALAT1 showed significant upregulation in neoplastic samples [69]. This finding laid the groundwork for a subsequent study in which plasma levels of MALAT1 were assessed in 88 HCC patients, 51 healthy controls, and 28 individuals with non-cancerous liver disease [70]. Circulating levels of MALAT1 were elevated in HCC samples (9.17 ± 43.62) compared to individuals with hepatic disease (1.10 ± 0.82) and healthy controls (0.85 ± 1.10). Interestingly, plasma MALAT1 levels in HCC patients with HBV infection were lower than those with HCV, and in those HCC patients with liver damage or cirrhosis, MALAT1 levels were substantially higher. ROC analysis between HCC patients and individuals with hepatic disease revealed a cut-off value of 1.60 and an AUC of 0.66. Sensitivity and specificity for the detection of HCC were highest (88.6% and 75%, respectively) using a combination of MALAT1, AFP, and DCP levels, compared to results using individual measures. Although these findings require additional validation, they provide preliminary evidence supporting clinical utility of plasma MALAT1 levels for predicting development of HCC and as a marker of liver damage. However, given the association of this lncRNA in many different kinds of cancer, the specificity of MALAT1 as a biomarker for HCC will need to be addressed.

### 3.3 UCA1

Kamel et al. [71] used in silico pathway enrichment analysis to identify urothelial carcinoma associated 1 (UCA1) as a potential candidate for carcinogenesis. Serum levels of UCA1 were significantly higher in HCC patients (N = 82) compared to chronic HCV patients (N = 34) and healthy individuals (N = 44) and were associated with age, smoking, HCV antibodies-positive patients, and Child-Pugh score. Serum UCA1 levels were also strongly correlated with expression levels in paired HCC tissue (r = 0.823; P < 0.01), suggesting that UCA1 may not only represent a biomarker for HCC, but may also play a role in carcinogenesis. In comparisons of HCC patients and healthy controls, the threshold of UCA1 was 1.04, with a sensitivity of 92.7%, indicating that the lncRNA could accurately distinguish HCC patients from individuals without cancer. In comparisons of HCC patients with chronic HCV patients, a value of 1.5 could discriminate the two groups, but with a reduced sensitivity and specificity. The area under the ROC was 0.861 for discriminating HCC patients from healthy controls, and 0.728 for discriminating HCC patients from chronic HCV patients. In an independent investigation comprised of 105 HCC patients, 105 individuals with benign liver disease (BLD), and 105 healthy volunteers, serum UCA1 levels were significantly higher in HCC patients compared with BLD patients or healthy controls [72]. ROC curve analysis demonstrated that serum UCA1 levels could distinguish HCC patients from healthy controls (AUC = 0.902) and at a cut-off threshold of 1.85; the sensitivity and specificity were 73.3% and 99.0%, respectively. Similar findings were obtained for comparisons of HCC and BLD patients. In HCC patients, elevated serum UCA1 levels were associated with high tumor grade, large tumor size, positive vascular invasion, and advanced TNM stage. In addition, the 5-year overall survival in HCC patients with high serum UCA1 levels was significantly worse than those with low serum levels. While these findings suggest that UCA1 may be a useful prognostic marker for HCC, other studies have found elevated plasma levels of this lncRNA in other kinds of cancer, suggesting that it may not be specific for HCC. However, given the evidence supporting association between serum UCA1 levels and clinicopathological features of HCC, investigation of this lncRNA in prospective studies with large sample sizes appears to be warranted.

## 4 Conclusions

The discovery of dysregulated lncRNAs has added a new layer of complexity to the molecular architecture of human disease. However, there are still many gaps in our current understanding of lncRNA function, and further study of these molecules is expected to yield deeper insights into mechanisms underlying the pathogenesis of many human diseases, development of new RNA-based targets for the prevention and treatment of disease, and improved methods for early detection of pathology.

Numerous studies have demonstrated a role for lncRNAs in multiple biological processes relevant to HCC, including the initiation, progression, metastasis, recurrence, treatment, and prognosis of the disease. Additional work has shown dysregulation of lncRNAs in tumor tissues is associated with these biological processes. Finally, a rapidly growing set of circulating lncRNAs is showing diagnostic and prognostic promise in HCC research. While the role of lncRNAs in HCC development and progression is still not completely understood, elucidation of the molecular mechanisms by which these noncoding transcripts affect disease pathogenesis will be critical for identifying potential targets for therapeutic development. Further investigation is expected to unravel the regulatory networks and molecular characteristics of lncRNAs, leading to improvements in the diagnosis, treatment, and possible prevention of HCC.
